# Life Employment Histories in Middle Age and Their Association With Health Outcomes in Later Life: A Sequence Analysis

**DOI:** 10.1093/geroni/igaa057.213

**Published:** 2020-12-16

**Authors:** Oejin Shin, BoRin Kim, Sojung Park, JiYoung Kang, Ilan Kwon, Seoyeon Ahn

**Affiliations:** 1 University of Illinois at Urbana Champaign, Savoy, Illinois, United States; 2 University of New Hampshire, Durham, New Hampshire, United States; 3 Washington University in St. Louis, St. Louis, Missouri, United States; 4 Hannam University, Daejeon, Republic of Korea; 5 Michigan State University, East Lansing, Michigan, United States; 6 National Pension Research Institute, Jeonjusi, Republic of Korea

## Abstract

Despite consistent evidence on the negative effect of precarious employment on health, little is known about the dynamic pattern of employment over the life course on later year health. Using the longitudinal data (1-18 waves) from the Korean Labor & Income Panel Study (KLIPS) (n=1,705), this study aimed to (1) identify long term change patterns of employment in two middle-age groups (early middle-aged: 40-49 / late middle-aged: 50-59), (2) examine the association between the patterns and self-rated health in old age. We apply sequence analysis with 18 years of working status and conducted regression health outcomes in the 18th wave. The result of sequence analysis found the differential employment patterns: among the early middle-aged, five patterns were identified consistently full-time, consistently not- working, transit to self-employed, mixed pattern, retired. Among the late middle-aged, five patterns were identified: consistently full time, mixed pattern, consistently retired, transit to not- working, consistently not working. Regression results indicated a negative association between precarious employment history and health in later years: Among the early middle-aged, members in the “consistently full-time” were likely to have better health compared to “transit to self-employed”. Among the later middle-aged, “retired” and “transit to not –working” were likely to have better health compared to “mixed pattern group”. The findings suggest the importance of employment history in middle age to predict the health outcome in later life. Policies to support experiencing precarious or self-employment are needed to prevent health disparity in later life.

